# Exploring the psychosocial challenges faced by pregnant teenagers in Ditsobotla subdistrict

**DOI:** 10.4102/hsag.v27i0.1880

**Published:** 2022-11-04

**Authors:** Peaceful N. Ntshayintshayi, Leepile A. Sehularo, Isaac O. Mokgaola, Nombulelo V. Sepeng

**Affiliations:** 1 Quality in Nursing and Midwifery (NuMIQ) Research Focus Area, Faculty of Health Sciences, North-West University, Mmabatho, South Africa; 2 Department of Nursing, University of Pretoria, Pretoria, South Africa

**Keywords:** pregnancy, psychosocial challenges, teenagers, unwanted pregnancy, parenthood, social exclusion

## Abstract

**Background:**

Pregnant teenagers usually experience psychosocial challenges such as a great amount of stress when they have to deal with an unwanted pregnancy, unpreparedness for parenthood and a lack of income as well as labour and birth complications. These are further complicated by the stigma from their families, friends and community. Unaddressed psychosocial challenges during teenage pregnancy can adversely affect the health outcomes of both mother and the child.

**Aim:**

This study explores and describes the psychosocial challenges faced by pregnant teenagers in the Ditsobotla subdistrict.

**Setting:**

The study was conducted in three health centres in the Ditsobotla subdistrict.

**Methods:**

A qualitative-exploratory-descriptive and contextual research design was used. Non-probability purposive and convenience sampling techniques were used to select the participants. Semistructured individual interviews through WhatsApp video calls were used to collect data, which were analysed using conventional content analysis.

**Results:**

Three themes emerged from the findings of the study, namely psychological challenges, social challenges and suggestions to address psychosocial challenges faced by pregnant teenagers.

**Conclusion:**

The findings established that pregnant teenagers in the Ditsobotla subdistrict are faced with psychosocial challenges which negatively impact their psychological health and social life. Suggestions made in this study have the potential to improve the psychosocial well-being of pregnant teenagers in the Ditsobotla subdistrict if implemented.

**Contributions:**

The findings of this study provide important information that may be used to improve the psychosocial well-being of pregnant teenagers in the Ditsobotla subdistrict.

## Introduction

Pregnancy occurring in girls between the ages of 13 and 19 years is a major health challenge worldwide, with the potential to adversely affect birth outcomes. This may also lead to ill health and a cycle of poverty (Abebe et al. 2020:1; Mann et al. 2020:312). A study by Walker and Holfreter (2021:299) indicates that teenage pregnancy is also a notable predictor of depression, alcohol and substance abuse. Wong et al. (2020:156) add that teenage mothers have a significantly higher rate of depression and anxiety during pregnancy than mothers aged 20–34 years. According to Gselamu et al. (2019:116), teenage mothers experience psychosocial challenges such as a great amount of stress before and after birth, arising from unwanted pregnancy and unpreparedness for parenthood. The same authors add that these teenage mothers also experience a lack of income as well as labour and birth complications taking the form of infant death, anaemia and low-birth-weight infants (Gselamu et al. 2019:116). According to Caffe et al. (2017:6), teenage pregnancy and motherhood affect physical health and pose the risk of social exclusion by family, friends and society. These complications and challenges severely affect the psychosocial well-being of teenage mothers. Parents, teachers and friends usually experience anger and disappointment when a teenager is pregnant, and their academic performance is also negatively affected or delayed (Quaye & Attom 2019:118). Unaddressed psychosocial issues before and during pregnancy can adversely affect the health outcomes of both mother and child (Harron et al. 2021:97). This information highlights why the researcher deemed it necessary to conduct this qualitative study.

In the United Kingdom (UK), a high prevalence of teenage pregnancy is observed in girls with risk factors such as being more likely to live in poverty, being unemployed or having lower salaries and educational achievements than their peers (Cook & Cameron 2017:327). In Indonesia, teenage pregnancy is associated with the stigma of engaging in extramarital sex; therefore, pregnant teenagers experience social, health and economic problems with little or no support from family and friends (Tjung, Serworwora & Yonathan 2021:232). According to Abeb et al. (2020:1), the lack of sexual education and cultural obedience are some of the contributing factors for teenage pregnancy in low- and middle-income countries (LMIC) such as Brazil, Bangladesh and Nigeria.

In the South African context, black Africans are the ethnic group with the highest percentage of teenage pregnancy of 12.5% and white people were the lowest with just 1.6% (Statistics South Africa 2018:25). In South Africa (SA), between 2018 and 2019, approximately 5% of girls between the ages 14 and 19 years were reported to have been pregnant in the past 12 months; the number of 19-year-old pregnant girls were 32 times higher. According to Mturi and Behuke (2019:20), in the Ngaka Modiri Molema District, particularly in Mafikeng in the North West province, young girls become pregnant out of ignorance or a lack of knowledge regarding sexuality. Therefore, it is important that sexual education in schools is introduced in lower grades. Teenage pregnancy in the North West province is one of the key causes of school dropout, and most girls who leave school due to pregnancy never return after childbirth (Masilo 2018:23). The above information emphasises the point that more attention needs to be focused not only on the physical well-being but also on the psychosocial well-being of pregnant teenagers. In spite of the above discussion, there are no studies on the psychosocial challenges faced by pregnant teenagers in the Ditsobotla subdistrict.

### Problem statement

Literature indicates that pregnant teenagers are faced with several psychosocial challenges which tend to negatively affect their psychosocial well-being (Mokhopadyay et al. 2004:495–497; Mokobocho-Mohlakoane 2005:3). According to the researcher’s personal experience as a professional nurse, some of these psychosocial challenges include stigmatisation, depression and suicide. The researcher noted that in the Ditsobotla subdistrict, the psychosocial well-being of pregnant teenagers during antenatal care (ANC) visits are, in most instances, neglected and more focus is on their physical well-being. In spite of the above challenges, there are no studies which have been conducted in the Ditsobotla subdistrict, which primarily focuses on exploring and addressing the psychosocial challenges faced by pregnant teenagers.

### Research aim

The aim of the study was to explore and describe the psychosocial challenges faced by pregnant teenagers in the Ditsobotla subdistrict.

### Research objectives

The objective of this study was to explore and describe the psychosocial challenges faced by pregnant teenagers in the Ditsobotla subdistrict. Another objective was to describe the suggestions to address the psychosocial challenges faced by pregnant teenagers in the Ditsobotla subdistrict, as perceived by pregnant teenagers.

### Research approach

A qualitative research approach was followed to achieve the aim and objectives of the study. Brink et al. (2016:121) indicate that a qualitative research approach is used when little is known about a phenomenon or when its context, nature, and boundaries are poorly defined and understood. In the Ditsobotla subdistrict, the psychosocial challenges faced by pregnant teenagers are not documented in the literature.

## Research methods and design

### Study design

A qualitative–exploratory–descriptive, and contextual research design was used in this study. Semistructured individual interviews were conducted through WhatsApp video calls, and the data was analysed using conventional content analysis.

### Study context

This study was conducted at three health care facilities in the Ditsobotla subdistrict, which is one of the four subdistricts of Ngaka Modiri Molema in the North West province. The population of the district is 48 651. There are three surrounding towns in Ditsobotla, namely Coligny, Lichtenburg and Biesiesvlei, which are surrounded by several farms and informal settlements where most of the people reside. The entire subdistrict is catered for by 18 clinics, including mobile clinics. All these clinics offer ANC services, and only nine caters for maternity (delivery). Amongst other health and social issues, residents in this subdistrict are faced with a burgeoning teenage pregnancy issue. Pregnant teenagers are seen at these clinics, and health centres, presenting with psychological and social issues which are often missed or left unattended.

### Population and sampling

The population was all pregnant women and teenage mothers between the ages of 13 and 19 years residing in the Ditsobotla subdistrict during the data collection period. Purposive and convenient sampling techniques were used by an independent person to select nine participants for the study. The independent person was a professional nurse with a Bachelor of Nursing Science degree. During the recruitment of participants, she was a Master of Nursing Science candidate at a local university and experienced in research. The independent person obtained permission to approach participants, who were below the age of 18, from their parents or guardians. Participants who were 18 and 19 years of age were considered adults and were able to give consent without the involvement of parents or guardians. Their telephone numbers were obtained by the independent person during the recruitment process. Sample size was determined by data saturation. Data saturation was reached after the ninth interview, when there was enough information to replicate the study, when the ability to obtain additional new information had been attained, and when further coding was no longer feasible (Fusch & Ness 2015:1408).

### Data collection

Informed consent was facilitated by the researcher and signed by the potential participants, their parents or guardians and their witnesses. Only participants who were 18 years and above signed their own consent forms. The researcher also had a witness. Nine semistructured individual interviews were conducted in English by the researcher using WhatsApp video calls, and the interviews lasted 30 min – 60 min. WhatsApp allowed the researcher to take field notes during the interview. Pilot testing was done to check the feasibility of the interview guide. A recording tape was used to record participants’ responses. Field notes were also used to record data which could not be obtained verbally. The following open-ended questions were asked:

1. What challenges did you encounter during your pregnancy?
2. In your opinion, what could be done to address those challenges?

### Data analysis

The researcher collected and transcribed data in preparation for data analysis. Conventional content analysis was used by the researcher and the co-coder to analyse data independently. Conventional content analysis is used in a study with the aim of describing a phenomenon, and it is the most appropriate method when there is limited literature and theory regarding the research phenomenon (Hsieh & Shannon 2005:1279). Researchers allowed new data to emerge and new themes to flow from the data. The steps of conventional content analysis in this study included identifying and collecting data, determining coding themes, coding the content, checking validity and reliability, analysing and presenting the results (Hsieh & Shannon 2005:1279).

### Ethical considerations

Ethical clearance was obtained from the North-West University Health Research Ethics Committee (NWU-HREC) (reference number NWU-00958-19-A1). Permission to approach the clinics was granted by the North West Provincial Department of Health (NWP-DoH) Ethics Committee. The managers of three health centres gave permission for the pregnant teenagers to be approached. The identity of participants was protected by the use of codes to refer to participants during data collection such as A, B, C and so on. Because of the sensitive nature of the topic of this study, a psychologist was on standby during data collection for participants experiencing emotional harm due to participation in this study. Information about participants was not shared with anyone outside of the research team. However, participants were informed that this study would be submitted for publication.

## Results

The results of this study are presented according to the demographic information, as well as the themes and subthemes.

### Demographic information of participants

Nine participants from three health centres participated in the study. The age of participants ranged between 15 and 19. Only three participants were pregnant, and six delivered. See Table 1 for more information.

**TABLE 1:** Demographic information of participants.

Participants	Age	Pregnant or delivered	Gestational age or babies’ age, 1 year and below	Educational level	Health centre no.
A	19 years	Delivered	8-month-old baby	Passed Grade12 (12th year of schooling)	Health centre 1
B	19 years	Delivered	12-month-baby	Grade 12 (12th year of schooling)	Health centre 1
C	16 years	Pregnant	6 months pregnant	Dropped out at Grade 9 (9th year of schooling)	Health centre 1
D	17 years	Delivered	3-month-old baby	Grade 10 (10th year of schooling)	Health centre 2
E	15 years	Delivered	3-month-old baby	Dropped out at Grade 9 (9th year of schooling)	Health centre 2
F	17 years	Delivered	1-month-old baby	Grade 10 (10th year of schooling)	Health centre 2
G	15 years	Pregnant	6 months pregnant	Grade 8 (8th year of schooling)	Health centre 3
H	19 years	Delivered	3-month-old baby	Dropped out at grade 10 (10th year of schooling)	Health centre 3
I	15 years	Pregnant	9 months pregnant	Grade 8 (8th year of schooling)	Health centre 3

**TABLE 2:** Themes and subthemes.

Themes	Subthemes
Theme 1: Psychological challenges	1.1. Experience of shock and anxiety 1.2. Experience of depressed mood 1.3. Poor coping mechanisms
Theme 2: Social challenges	2.1. Teenagers felt that they disappointed family and the community 2.2. Stigma from family members 2.3. Social withdrawal and rejection by the community 2.4. Nurses’ negative attitude towards teenage pregnancy 2.4. Experiences of financial constraints 2.5. Rejection by friends and partners
Theme 3: Suggestions to address the challenges faced by pregnant teenagers	3.1. Health education programmes for teenagers 3.2. Health awareness campaigns for community members 3.3. Provision of family planning services in schools

Three themes emerged from the findings of the study, which are psychological challenges, social challenges and suggestions to address the challenges faced by pregnant teenagers. Themes and subthemes are amplified in the following sections.

### Theme 1: Psychological challenges

Three subthemes emerged from the psychological challenges: experience of shock and anxiety, experience of depressed mood and poor coping mechanisms. These subthemes and participants’ quotations are given in the following sections:

#### Experience of shock and anxiety

Most participants voiced that they became shocked and anxious when they found out about their pregnancy. Participants stated the following:

‘OK, first of all, I was shocked and overwhelmed, mainly because my parents are too strict, and they emphasised that if ever I fall pregnant early, I will see where I go with my baby or how I will take care of my baby, because it was none of their business. They said I’ll have to deal with my issues and stuff like that so … I was very terrified and … It came as a shock because I didn’t expect that it was going to happen that soon … yes.’ (Health Centre 2, Participant E, 15 years)

‘I had a lot of stress at first. I thought of having an abortion and then I was scared … even worse at the idea of taking my life…’ (Health Centre 2, Participant F, 17 years)

#### Experience of depressed mood

Participants mentioned that after discovering that they were pregnant, they experienced depression and other unpleasant feelings. Some even contemplated suicide. This is what some of the participants said:

‘I was just, I don’t know … I think I was just experiencing a lot of family problems and which led me to several suicide attempts. I was admitted in a mental health care facility for depression … and I tried several times to take my life.’ (Health Centre 1, Participant B, 19 years)

‘With emotions … you know … with pregnancy there are moments where you feel like you regret everything and wish that you could have somebody to talk to … somebody that understands you … So …I didn’t have that somebody so … in most cases, I think that it brought me to depression because I remember I would touch my tummy and wish that there could be a way I could get rid of the baby.’ (Health Centre 2, Participant E, 15 years)

#### Poor coping mechanisms

Some of the participants explained how coping with teenage pregnancy was difficult. Some of the participants mentioned that they thought they would not survive to see the delivery of the pregnancy. To confirm this finding, participant said:

‘Given that I am a student, it’s very difficult to cope with the pregnancy and kafa; there’s a lot of work that I needed to do.’ (Health Centre 1, Participant A, 19 years)

‘[…*W*]hen I mean that it was difficult to cope, my baby was a premature and I didn’t think that she would survive, so it was very painful; it was a very difficult time. The whole pregnancy, the whole 7 months, it was just, jah, it wasn’t easy at all.’ (Health Centre 1, Participant A, 19 years)

‘I wasn’t … I don’t know, I just felt like I wasn’t ready for motherhood. I just kept asking myself, how am I going to be a mother? Because I also had to go to matric and the stress and pressure that comes with being in matric and stuff like that.’ (Health Centre 1, Participant B, 19 years)

### Theme 2: Social challenges

Six subthemes emerged from the social challenges, namely that teenagers felt that they disappointed family and the community, stigma from family members, social withdrawal and rejection by the community, nurses’ negative attitudes towards teenage pregnancy and experiences of financial constraint as well as rejection by friends and partners. Participants’ verbatim submissions are given below to support these subthemes:

#### Teenagers felt that they disappointed family and the community

Participants felt that their pregnancy brought shame and disappointment in their family. These are some of the quotations from the individual interviews:

‘My aunt and my grandma were hurt. They felt so disappointed, they were not expecting that at this time I would be pregnant, as they were looking up to me and they couldn’t understand when I fell pregnant while I always slept at home every night. They were very disappointed in me.’ (Health Centre 3, Participant H, 19 years)

‘They were ashamed; they didn’t expect gore, it wasn’t what was, what they expected from me, knowing that I was young and a student, they didn’t expect that I will be pregnant.’ (Health Centre 1, Participant A, 19 years)

#### Stigma from family members

Some participants mentioned that they were stigmatised by their family because of the pregnancy. The following quotations endorse this interpretation of what the participants said:

‘My brothers … it’s been … all these people talking about stigma and people looking at you badly and in different ways. I thought it was a joke until I had to experience it … it was, yoh … it was bad it wasn’t nice … jah.’ (Health Centre 2, Participant D, 17 years)

‘My family stigmatised me for being pregnant, that’s what I hated the most about my pregnancy.’ (Health Centre 3, Participant I, 15 years)

#### Social withdrawal and rejection by the community

Some of the participants mentioned that teenage pregnancy brought a massive change to their social life. Participants mentioned that they experienced rejection from the community. To confirm this finding, two of the participants expressed the following vignettes:

‘Yes, I’ll like to talk about the discrimination that happens in churches. You might find that you were on the worship team, and they have to cut you off because they believe that you are a wrong example to other kids. I understand that there are rules in every church, but they must not be rude. They can just call you aside and tell you that you must step down and take care of the baby and you will come back. Instead of making you feel like an outcast and that you are a bad example to other kids. Even though at church you are supposed to feel safe, but that is where you are judged the most. I wish there could be something that can be done about that too.’ (Health Centre 2, Participant E, 15 years)

‘I liked being indoors; besides that, I didn’t want people to see me.’ (Participant A, 19 years)

#### Nurses’ negative attitude towards teenage pregnancy

Participants in this study mentioned that the nurses have negative attitudes towards teenage pregnancy. They further mentioned that nurses made their lives difficult when they were pregnant. To confirm this finding, one of the participants said:

‘Oh, also, at the clinic, the nurses were giving me a lot of attitude because most of the time I would ask for permission from the principal to go to the clinic. … So the nurses gave me very … very bad words. They told me very … very painful things, that I must stop sleeping around with boys and I must focus on my school. None of them gave me that acknowledgment that I am pregnant.’ (Health Centre 2, Participant D, 17 years)

‘Yes, like at the clinics, you see, when you get there and you are young, they don’t treat you the right way … it’s like an embarrassment. At least if they taught us about teenage pregnancy and how to avoid it instead of shaming us, even at school or in the clinics, you see.’ (Health Centre 2, Participant F, 17 years)

#### Experience of financial constraints

Pregnant teenagers experienced financial struggles during their pregnancy. Financial adjustments were made in the family to cater for the new child’s needs. Two confirmed this finding in submitting their own experiences:

‘Eh, acceptance in the family … [*was*] also the biggest challenge I was facing. This pregnancy alone … because now my mother is not able to take care of all my needs, and now my school uniform doesn’t fit me anymore, and my mother cannot go to her job.’ (Health Centre 3, Participant G, 15 years)

‘[…*T*]hey are still worried how we are going to survive now with an extra mouth, but it’s not as bad as it was before.’ (Health Centre 3, Participant G, 15 years)

‘Most of the challenges would be that financially, my parents had to cut me off from the money they used to give me. They specified that the money they were giving me is going to be used for my baby. Now I am cut off from many privileges financially… so … jah … it’s just a messed-up situation.’ (Health Centre 2, Participant E, 15 years)

#### Rejection by friends and partners

Most pregnant teenagers are rejected and rebuffed by their friends and partners after finding out about their pregnancy. These participants mentioned that everything was fine before teenage pregnancy. To confirm this finding, two of the participants said:

‘Jah, I just felt like I was alone even though some of my friends were there. I felt like I needed my boyfriend to also be there to support me … because he was moving on and he had another girlfriend, so it was not great for me.’ (Health Centre 1, Participant B, 19 years)

‘[…*M*]y boyfriend at the time got very weird when I told him the news. He told me that I’ve been sleeping around, he doesn’t have a baby with me and stuff like that … so right now we are not together anymore. I am taking care of the baby alone with my family.’ (Health Centre 2, Participant D, 17 years)

### Theme 3: Suggestions to address challenges faced by pregnant teenagers

Three subthemes emerged from the suggestions submitted by the participants to address the challenges faced by pregnant teenagers, namely health education programmes for teenagers, health awareness campaigns for community members and the provision of family planning services in schools. The following responses were provided by the participants, and they are interpreted as evidence of the themes stated above:

#### Health education programmes for teenagers

Participants in this study mentioned that health education programmes may be an effective way to educate and alert other teenagers about the challenges arising from teenage pregnancy and equip them with some knowledge to prevent this unnecessary teenage burden. This subtheme is supported by the following quotations:

‘I think teenagers should be educated on how to protect themselves to avoid teenage pregnancy, and it shouldn’t only be focused on the girls because most of the time girls are told that you should prevent, and you should do this and that, but this must be focused to both girls and boys.’ (Health Centre 1, Participant B, 19 years)

‘The school … they can give us better advice to us in terms of family planning … maybe they can include the factor of explaining further about family planning and explaining how it can affect you in your family, not just pregnancy and finances. They must also explain that I can also bring tension into the home.’ (Health Centre 1, Participant B)

#### Health awareness campaigns for community members

Participants interviewed in this study mentioned that health awareness campaigns in the community may be an effective method to enlighten the community about the psychosocial challenges pregnant teenagers face and consequently reduce the stigma and rejection. Participants expressed the following:

‘Something could be done at the community level for them to understand teenage pregnancy so that they stop saying horrible things when they see a pregnant teenager.’ (Health Centre 2, Participant F, 17 years)

‘And … OK, it happened. I think the community must not be so discriminating towards us, since it adds on to the stress, and I will learn from my mistakes, and even my family, they must understand that it was a mistake, and I will do better next time. Even the teachers, they should not act like that, because if they do this, they are encouraging the learners to bully me.’ (Health Centre 3, Participant G, 15 years)

#### Provision of family planning services in schools

Participants interviewed in this study mentioned that there was a need for family planning services to be provided in schools. These participants further mentioned that the majority of teenage pregnancy cases occur amongst school-going children. The following quotations support what the participants said:

‘I don’t know if the nurses and the doctors can come to school and give us the injections to stop the pregnancy. Maybe they can also create something for the boys as well because, yoh, there’s one boy in my class … he has five kids, imagine … so maybe that can help too.’ (Health Centre 2, Participant D, 17 years)

‘I think … firstly, I admit that I am too young to fall pregnant, and maybe I should have used prevention and I wouldn’t be in this situation.’ (Health Centre 3, Participant G, 15 years)

### Measures of Trustworthiness

Credibility, dependability, confirmability and transferability, as explained by Burns and Grove (2011:38), were used to ensure the trustworthiness of this qualitative study. Credibility were achieved by ensuring that data were collected through semistructured interviews through WhatsApp video calls until saturation was reached. To ensure transferability of this study, the research methodology and sampling technique were described in depth to ensure transferability of this study. A small sample size of nine participants was used in this study. Therefore, the results of this qualitative study cannot be generalised but can be applied to other districts and subdistricts of SA. Confirmability of the study was achieved through the collection of data, using semistructured individual interviews through WhatsApp video calls, together with several data collection tools such as voice records and written field notes. Findings of both the researcher and the independent co-coder were compared to avoid researcher bias. Both the researcher and the independent co-coder reached a consensus on the final themes and subthemes. Dependability was ensured through thick description of the research methodology utilised, literature control and thick description of the data analysis method in terms of data transcription, forming of codes and adequately describing them.

## Discussion

The aim of this study was to describe, explore and address the psychosocial challenges faced by pregnant teenagers in the Ditsobotla subdistrict. Three themes were generated: psychological challenges, social challenges and suggestions to address the challenges faced by pregnant teenagers. These themes and subthemes were supported by the participants’ verbatim quotations.

Psychosocial challenges emerged as the first theme in this study, with three subthemes, which were that pregnant teenagers experience shock and anxiety. Most women with unplanned pregnancies tend to react negatively to the news of their pregnancy, to a point that some even opt for abortion (White, Mann & Larkan 2017:473). The fear of consequences amongst pregnant teenagers tends to delay them from attending ANC (Molokwane et al. 2018:26). On the other hand, Zainudin et al. (2019:34) mention that teenagers had to make sense of their experience, where some expressed great feelings of regret, remorse and guilt for engaging in sexual relationships well after finding out about their pregnancy. Pregnant teenagers present with depressed mood. There is a significant relationship between teenage pregnancy and long-term mental health impact (Xavier et al. 2018:455). The additional burden and social risks that come with teenage pregnancy predispose pregnant teenagers to mental illnesses such as depression and anxiety (Dahmen et al. 2019:247). There is apparently an increase in co-occurrence of mental conditions and teenage pregnancy such as anxiety, depression and suicidal ideation (Doghor et al. 2019:70). Jalanko et al. (2020:348) also emphasised that adolescent pregnancy poses an increased risk of psychiatric morbidity in adulthood, regardless of whether it ended as childbirth or abortion.

Pregnant teenagers who lack resilience are at risk of depression and suicide, as they may not be able to cope with the physical and emotional discomfort associated with pregnancy (Kausit 2020:830). In this study, participants verbalised that they would prefer to stay indoors during their pregnancy to avoid being seen and mocked. Poor coping mechanisms also emerged as one of the subthemes. Mokoena (2018:60) also established that during pregnancy, teenagers sometimes adopt ineffective coping mechanisms, whilst others develop behavioural patterns to cope. Social media was one of the platforms that they use to cope with the current situation. For instance, pregnant teenagers would be spending more time on social media rather than attending to their pregnancies. Other poor coping mechanisms adopted by pregnant teenagers included relocating to another area, handing the baby over to another person to nurse and considering or having an abortion, whilst some employed positive coping mechanisms such as personal resolutions and external encouragement (Ashimolowo et al. 2017:15). The above discussion highlights that there is a need for further studies addressing the psychological challenges experienced by pregnant teenagers.

Social challenges also emerged as one of the main themes in this study. Under this theme, teenagers felt that they disappointed their family and the community. In fact, the pregnant teenagers in this study also experienced stigma from family members. According to Masilo (2018:40), the impact of teenage pregnancy as a social problem is not only felt by the pregnant teenagers themselves but also the family, school and society. Parents normally experience mixed emotions about their teenage girl’s pregnancies, and their reactions are influenced by their norms and beliefs (Sriyasak et al. 2018:41). Tambi and Mesue (2020:36) state that teenagers experience rejection from parents and are also provoked at school. Parents may feel disappointed and ashamed when their teenage daughter is pregnant. Some even scream or use harsh words. Counselling for parents of pregnant teenagers may help them to be supportive of their daughters, as the pregnancy affects both the parents and the teenage girl (Tambi & Mesue 2020:38). This is also especially important to the mothers who experienced teenage pregnancy themselves. Social withdrawal and rejection by community members were raised by participants as some of the social challenges they faced. According to Chirwa Msuku (2016:47), due to their early childbearing, pregnant teenagers experience rejection, stigmatisation, social isolation and mockery from their families, friends and society. The social isolation and stigma from the community generally drive some of the causes of suicide amongst pregnant teenagers (Musyimi et al. 2020:20).

Nurses’ negative attitudes towards teenage pregnancy were also mentioned as challenges encountered by participants of this study. Jonas et al. (2017) state that the influence of social norms and beliefs amongst health care providers still hinders proper and adequate provision of reproductive health services to adolescents. According to Onokerhoraye and Dudu (2017:88), pregnant teenagers experience judgemental attitudes, lack the essential confidentiality and experience unsatisfactory service delivery which inhibit pregnant teenagers from accessing reproductive health services. Jonas et al. (2017:14) also state that the attitude and behaviour of health care workers may affect the provision of sexual health services such as ANC to pregnant adolescents.

Participants experienced financial constraints during teenage pregnancy. Early motherhood has significant negative effects on adolescent mothers, and this lack of much-needed social support during their pregnancy becomes a serious hurdle. This impedes the entire process of raising their babies in such an economically strained context (David, Van Dyk & Ashipala 2017:45). Rejection by friends and partners was raised by participants as another of the social challenges pregnant teenagers face. According to Ellis-Sloan and Trampling (2019:210), friendship is undermined as a form of support system. Adolescent pregnancy has the potential to destroy friendships and leave the pregnant teenager isolated and lonely. According to David et al. (2017:42), adolescent mothers are faced with distorted interpersonal relationships with friends, family and partners.

In spite of the challenges cited above, participants suggested practices and protocols that may be used to address the challenges faced by pregnant teenagers. In this case, participants mentioned that there should be health education programmes for teenagers and health awareness campaigns for community members. De Wet, Amoo and Odimegwu (2018:49) recommend that with a specific focus on the involvement of young men, there should be initiatives to create awareness about teenage pregnancy amongst the youth. Youth support groups and health programmes if properly implemented could help curb the stigma towards young pregnant girls (Chirwa Msuku 2016:43).

Participants in this study voiced that the provision of family planning services in schools may help minimise the number of girls falling pregnant. There is very limited content in the South African school sexuality education curriculum, and it is these restrictions that complicate matters regarding age, as pregnancy occurs in girls as young as 13 years (Mturi & Bechuke 2019:140). A significant number of adults support the introduction of sex education to the middle and high school curriculum with topics such as sexual orientation, contraceptives, sexually transmitted infections (STIs) and many others (Kantor, Levitz & Holstrom 2020:242). Nurses indicate that they get limited access to schools to provide reproductive health and sex education and also provide family planning services to adolescents, which they believe would be effective in addressing the high number of pregnant teenagers (Jonas et al. 2017:11).

### Limitations

The study was conducted in three community health centres in the Ditsobotla subdistrict and cannot be generalised to other community health centres in the NWP or SA. The only limitation of the WhatsApp data collection method was network challenges with one of the participants. The researcher had to call many times to complete the interview.

### Recommendations

Further research is recommended on teenage pregnancy in the Ditsobotla subdistrict. Such research should use different methods such as quantitative and mixed methods which could possibly assist in generalisation of the findings. Such studies may focus on strategies to address those challenges experienced by pregnant teenagers. It is further recommended that family planning services be included in schools as participants mentioned that the majority of them fell pregnant whilst in school.

## Conclusion

The findings of this study show that pregnant teenagers encounter and experience psychological and social challenges in their daily lives, and those challenges pose a negative effect on their well-being in the Ditsobotla subdistrict. Those challenges include experiences of depressed mood, rejection by friends and partners, financial constraints and negative attitudes by nurses towards teenage pregnancy. Suggestions made to reduce the stigma from the community and families involve health awareness campaigns, health education programmes focused on teenagers and the availability of family planning education and methods in schools for teenagers.
